# Damage Detection of Bridges under Environmental Temperature Changes Using a Hybrid Method

**DOI:** 10.3390/s20143999

**Published:** 2020-07-18

**Authors:** Xiang Wang, Qingfei Gao, Yang Liu

**Affiliations:** 1 China Railway Bridge Science Research Institute, Ltd., Wuhan 430034, China; wangxiang4143@163.com; 2 State Key Laboratory for Health and Safety of Bridge Structures, Wuhan 430034, China; 3 School of Transportation Science and Engineering, Harbin Institute of Technology, Harbin 150090, China; ly7628@hit.edu.cn

**Keywords:** damage detection, bridges, time-varying temperature, principal component analysis, Gaussian mixture method

## Abstract

Principal component analysis (PCA)-based method is popular for detecting the damage of bridges under varying environmental temperatures. However, this method deletes some information when the damage features are projected in the direction of nonprincipal components; thus, the effectiveness of PCA-based methods will decrease if the deleted information is related to bridge damage. To address this issue, a hybrid method is proposed to detect the damage of bridges under environmental temperature changes. On one side, the PCA-based method is applied to deal with the nonprincipal components; on the other side, the Gaussian mixture method (GMM) is used to classify all the principal components into different clusters, and then the novel detection method is implemented to detect bridge damage for each cluster. In this way, all the damage feature information is saved and used to detect bridge damage. The numerical example and example of an actual bridge show that the proposed hybrid method is effective in detecting bridge damage under environmental temperature changes. The GMM is effective for classifying the natural monitoring frequency data of actual bridges, and the relationship between the natural frequencies of actual bridges and the environmental temperature is not always linear.

## 1. Introduction

Using the advanced sensing technique, structural health monitoring (SHM) technique can diagnose the structural damage and assess the structural safety of bridges by using the different types of structural response [1,2,3,4]. The core mission of SHM is to detect potential damage of bridges; thus, some methods for damage detection have been proposed [5,6,7,8], and among them, vibration-based approaches have shown excellent potential. Bridges inevitably suffer from the actions caused by varying environmental temperatures; furthermore, the abovementioned actions may mask the changes in damage features—e.g., the natural frequencies of bridges—caused by structural damage. Sohn et al. found out that the natural frequencies of the Alamosa Canyon Bridge changed about 6 percent per day with the variation of environmental temperature [9]. Farrar et al. investigated the change of the natural frequencies of the I-40 Bridge, the results showed that the serious artificial damage occurred in this bridge only caused little change of the natural frequencies [10]. The similar research results were obtained in references [11,12], and the results showed that the changing of natural frequencies caused by the structural damage of the Z24 Bridge in Switzerland was less than the variation of the natural frequencies induced by the environmental temperature changes. Therefore, some vibration-based methods, especially data-driven methods, focus on mitigating the influence of environmental temperatures on the results of damage detection for bridges.

One type of data-driven algorithm [13,14,15,16,17] focuses on establishing the relationship between the environmental temperature and damage features first, and then the generated model is applied to eliminate the effects of the environmental temperature. Additionally, the relationship between the environmental temperature and damage features of bridges is not always close to linear. Although some metamodel-based methods [18,19,20,21,22] is investigated to establish the abovementioned relationship, it is difficult to obtain an accurate relationship model, which may decrease the sensitivity of the method for structural damage assessment. Another type of data-driven diagnostic algorithms [23,24,25] do not require environmental temperature data at all, and the effects of environmental temperature on damage features are described as latent variables during the entire process of damage detection.

Among these methods, PCA [26] is widely adopted for detecting the damage of structures by using the data obtained from SHM system. With the orthogonal decomposition technique, PCA is proper to establish the baseline statistical model (pattern) using the measured data of structures under the healthy condition, and then the damaged condition of structure could be detected by utilizing the pattern recognition techniques [27,28]. Using this idea, PCA has been applied to detect the nonlinearity effects of structural integrity of an offshore structure [29], diagnose the abnormal condition of composite structures [30], and monitor the stress conditions of cylindrical specimens using PZTs [31]. Additionally, PCA only needs the simple calculation process, so it also has been adopted for detecting the damage of structures under the environmental temperature changes. Koo et al. proposed a temperature-free damage index based on PCA [32] by using the impedance information of structures. Bellino et al. verified that PCA is effective to detect the damage of both the linear time-invariant systems and the linear time-varying system [33]. Giraldo et al. proposed a method to localize the damage in the structure regardless of the environmental conditions [34]. Reynders et al. proposed kernel PCA based method to eliminate environmental influence for damage detection of structures [35]. Zhu et al. presented a temperature-driven moving PCA method to separate the thermal-induced response and detect the abnormal condition of structures [36].

The method proposed by Yan et al. [37,38] is a representative and effective PCA-based method to mitigate the influence of environmental temperatures on the results of damage detection of structures. The basic concept of this PCA-based method is to generate the covariance matrix of damage features first, and then all the damage features are projected in the direction of the nonprincipal component of the abovementioned covariance matrix. All these projected damage features are believed to be uncorrelated with the effects of environmental temperature variations because the direction of the principal component represents the influence of the environmental temperature. Next, the novelty detection [39,40] is applied to detect bridge damage by using the projected damage features. As described above, with the PCA-based method, some information is removed when the damage features are projected in the direction of nonprincipal components. If this deleted information is related to bridge damage, the effectiveness of the PCA-based method will weaken. Similar conclusions were also found in [41].

To overcome the abovementioned issue, a hybrid method which combines the PCA-based method and Gaussian mixture method (GMM) [42,43] is proposed in this study. GMM is a density model comprising a number of Gaussian distribution components, so GMM also is a method for the cluster analysis. PCA combined with GMM has been carried out for several fields such as speaker identification [44,45], tracing of moving objects [46], process monitoring of industry chemical processes [47], flood damage detection [48], fault diagnosis [49], etc. In this study, the combination between PCA and GMM is being applied for the first time to detect the damage of bridges under changing environmental temperature. With the proposed hybrid method, all the damage features are simultaneously projected in the direction of the principal components and the direction of the nonprincipal components. The PCA-based method is applied to address the nonprincipal components. For the damage features projected in the direction of the principal components, the GMM is utilized to classify all the projected damage features into different clusters. For each cluster, all the projected damage features satisfy the Gaussian probability distribution; thus, the novelty detection based on Gaussian probability distribution is implemented to detect bridge damage. Using this approach, all the damage feature information is saved and used to detect the damage of bridges.

In this context, the layout of this paper is as follows. In Section 2, the details of the proposed hybrid method are described. A numerical example is used to compare the damage detection performance of the PCA-based method and the proposed hybrid method in Section 3. In the next section, the natural frequency data collected for an actual bridge are utilized to verify the effectiveness of the proposed method. Finally, the conclusions are drawn.

## 2. Hybrid Method for Damage Detection of Bridges under Environmental Temperature Changes

In this section, the PCA based method is briefly reviewed, and then the issue of weakening the effectiveness of this method for detecting damage of bridges is proposed. To address this issue, the novelty detection method combined with GMM cluster analysis is presented to address the main components of damage features, which are introduced in detail. Finally, the procedure of the proposed hybrid method is described.

### 2.1. Discussion of the Effectiveness of the PCA-Based Method for Damage Detection of Bridges

Different response information for a structure can be used to establish damage features and detect the damage of bridges. For convenient description, the natural frequency monitoring data for a bridge are adopted to establish the damage features in this study. Assuming that n modes of natural frequencies are observed for a bridge, the matrix of natural frequency monitoring data, i.e., the damage feature matrix, is generated as

(1) $$f=[f_{1}^{},f_{2}^{},\cdots ,f_{v}^{},\cdots ,f_{m}^{}{]}_{n\times m}\;\left(v=1,2,\cdots ,m\right),$$

where $f_{v}^{}={\left\{f_{v1},f_{v2},\cdots ,f_{vi},\cdots ,f_{vn}\right\}}^{T}\left(i=1,2,\cdots ,n\right)$ is the vector of natural frequencies at the *v*th sampling time and m is the total number of monitoring samples. According to statistical theory, the sample mean and covariance of f are defined as the following vector and matrix, respectively

(2) $$\bar{f}=\left\{E\left(f\right)\right\}=\frac{1}{m}\sum_{v=1}^{m}f_{v},$$

(3) $$\Sigma =\frac{1}{m}\sum_{v=1}^{m}\left(f_{v}^{}-\bar{f}\right){\left(f_{v}^{}-\bar{f}\right)}^{T},$$

with Equation (3), the covariance matrix of f is obtained by using the natural frequency monitoring data. The above generated covariance matrix can be divided into three parts by using the technique of singular value decomposition (SVD). The decomposition of the covariance matrix is carried out as

(4) $$\Sigma =USV^{T},$$

where S is the singular value matrix, which is a diagonal matrix, and U and V are the singular vector matrices. The singular value matrix is defined as

(5) $$\begin{array}{l} S=\left[\begin{array}{cc} S_{1} & 0\\ 0 & S_{2} \end{array}\right]\\ S_{1}=\mathrm{diag}\left(s_{1}^{},s_{2}^{},\cdots ,s_{r}^{}\right)\\ S_{2}=\mathrm{diag}\left(s_{r+1}^{},s_{r+2}^{},\cdots ,s_{n}^{}\right) \end{array}$$

where diag(·) represents the diagonal matrix and $s_{r}^{}$ is the *r*th normalized singular value. All the singular values are arranged in descending order.

For the PCA-based method, the larger a singular value is, the more information the component possesses; thus, the singular values are applied to determine the principal components and nonprincipal components. As described in Equation (5), the matrices $S_{1}$ and $S_{2}$ represent the singular values corresponding to the principal components and nonprincipal components, respectively. Correspondingly, the singular vector matrix U is divided into two parts as

(6) $$U=[\begin{array}{cc} U_{1} & U_{2} \end{array}],$$

where $U_{1}$ consists of the first r column vectors of matrix U and $U_{2}$ is composed of the last n−r column vectors of matrix U.

**Figure 1:**
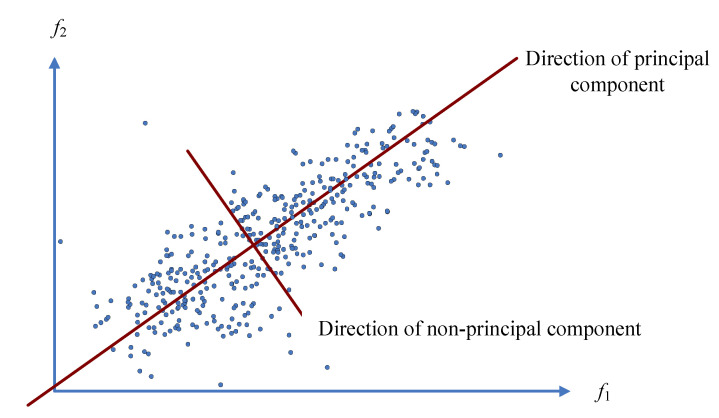
Schematic diagram of the PCA-based method with a two-dimensional example.

For convenient description, an example with two-dimensional damage features is used to express the basic concept of the PCA-based method. As shown in Figure 1, each sample point is obtained by using two damage features, $f_{1}$ and $f_{2}$, and it is obvious that there are two directions among all the damage features. The direction of the principal components represents the main trend of the damage features, and for a healthy bridge, this trend is determined by the effects of environmental factors such as the environmental temperature. Conversely, the direction of the nonprincipal components is orthogonal to the direction of influence of the environmental temperature, so all the damage features are projected in this direction to alleviate the effect of the environmental temperature. Specifically, the damage features projected in the direction of nonprincipal component $\theta_{2}$ are obtained by the equation

(7) $$\theta_{2}=U_{2}^{T}f,$$

After obtaining $\theta_{2}$, the probability distribution of $\theta_{2}$ for a healthy bridge state is generated by statistical analysis. With the generated statistical characteristics of the probability distribution, the Mahalanobis distance is applied to calculate the discriminant metrics of $\theta_{2}$, and then the threshold is established by using all the discriminant metrics. When the damage features of the state to be diagnosed are obtained, with Equation (7), the damage features are projected in the direction of the nonprincipal components generated using the monitoring data in the healthy state, which are defined as $\theta_{2}^{*}$. The discriminant metric of $\theta_{2}^{*}$ is calculated in the same way as $\theta_{2}$. If the values of a discriminant metric is larger than the established threshold, the bridge is considered to be damaged.

With the abovementioned approach, the PCA-based method is commonly applied to mitigate the influence of fluctuating environmental temperatures on the results of damage detection of bridges. The key of the PCA-based method is to utilize the information projected in the direction of the nonprincipal components, i.e., $\theta_{2}$ and $\theta_{2}^{*}$, and all this information is believed to be uncorrelated with the effects of varying environmental temperatures and mainly measurement noise. When structural damage occurs to a bridge, $\theta_{2}^{*}$ includes some structural damage that does not satisfy the probability distribution of $\theta_{2}$, so the damage will be detected successfully.

For the healthy state of bridge, the damage features projected in the direction of principal component $\theta_{1}$ are obtained by the equation

(8) $$\theta_{1}=U_{1}^{T}f,$$

Similarly, for the state to be detected for damage, the damage features projected in the direction of the principal components $\theta_{1}^{*}$ can be obtained. In theory, when damage occurs to a bridge, the information related to damage should be included in all the components of the damage features, i.e., $\theta_{1}^{*}$ and $\theta_{2}^{*}$. When $\theta_{2}^{*}$ includes the information related to structural damage, the PCA-based method works well for detecting bridge damage under varying environmental temperatures if the difference between $\theta_{2}$ and $\theta_{2}^{*}$ caused by damage is larger than the effects of measured noise included in $\theta_{2}$. When $\theta_{2}^{*}$ does not have any information related to structural damage, the PCA-based method cannot detect the structural damage, regardless of how serious the bridge damage is. Unfortunately, we cannot know in advance when and where the structural damage will occur; thus, it cannot be assured that $\theta_{2}^{*}$ includes information related to the structural damage. Accordingly, the PCA-based method may weaken the effectiveness of bridge damage detection in some cases. To address this issue, we proposed a hybrid method to simultaneously use all the information included in $\theta_{1}^{*}$ and $\theta_{2}^{*}$. The PCA-based method is utilized to deal with the nonprincipal component information, and the novel detection method combined with GMM cluster analysis is applied to deal with principal component information. In this way, all the information related to structural damage is saved and used to detect bridge damage.

### 2.2. Classification of the Damage Features Projected in the Direction of Principal Components Using GMM

As discussed in the above section, the Mahalanobis distance based on a Gaussian probability distribution is applied to calculate the discriminant metrics of $\theta_{2}$; however, for the damage features projected in the direction of principal component $\theta_{1}$, a Gaussian probability distribution is not valid because the environmental temperature is nonstationary. To address this issue, the GMM is used to classify $\theta_{1}$ into several clusters, and for each cluster, the components of $\theta_{1}$ satisfy the Gaussian probability distribution. Therefore, the novel detection approach based on the Mahalanobis distance is implemented for every cluster.

After obtaining $\theta_{1}$, combined with the environmental temperature monitoring data, the following matrix is defined as

(9) $$\begin{array}{l} \Xi ={\left[T,{\left[\theta_{1}\right]}^{T}\right]}_{m\times \left(r+1\right)}\\ T={\left\{T_{1},T_{2},\cdots ,T_{m}\right\}}^{T}\\ \theta_{1}=[\theta_{11}^{},\theta_{12}^{},\cdots ,\theta_{1g}^{}\cdots ,\theta_{1r}^{}{]}^{T},\left(g=1,2,\cdots ,r\right) \end{array}$$

where the vector $\theta_{1g}^{}$ consists of *m* terms, e.g., $\left\{\theta_{1g,1},\theta_{1g,2},\cdots ,\theta_{1g,m}\right\}$, and T represents the environmental temperature monitoring data. Ξ is sampled from a (r+1) dimensional continuous random distribution, and the density of the abovementioned probability distribution F(Ξ) is estimated by the following equation.

(10) $$F\left(\Xi_{v}\right)=\sum_{\beta =1}^{\alpha }\tau_{\beta }\Phi_{\beta }\left(\Xi_{v}|\mu_{\beta },\sigma_{\beta }\right),$$

where α represents the total number of clusters; $\tau_{\beta }$ is the mixing proportion of the βth cluster ($\sum \tau_{\beta }=1$). $\Phi_{\beta }\left(\Xi_{v}|\mu_{\beta },\sigma_{\beta }\right)$ is the multivariable Gaussian density for the βth cluster with mean $\mu_{\beta }$ and covariance $\sigma_{\beta }$, which is defined as

(11) $$\Phi_{\beta }\left(\Xi_{v}|\mu_{\beta },\sigma_{\beta }\right)=\frac{1}{\sqrt{{\left(2\pi \right)}^{r+1}\left|\sigma_{\beta }\right|}}\exp\left[-\frac{1}{2}\left[\Xi_{v}-\mu_{\beta }\right]{\left[\sigma_{\beta }\right]}^{-1}{\left[\Xi_{v}-\mu_{\beta }\right]}^{T}\right],$$

Expectation maximization (EM) algorithm is a common way to estimate the parameters defined in Equations (10) and (11) by using the following two steps.

For the first step, the following posteriori probability is obtained by given $\tau_{\beta }$, $\mu_{\beta }$, and $\sigma_{\beta }$ of the βth cluster

(12) $$\gamma_{\xi \beta }=\frac{\tau_{\beta }\Phi_{\beta }\left(\Xi_{v}|\mu_{\beta },\sigma_{\beta }\right)}{\sum_{\beta =1}^{\alpha }\tau_{\beta }\Phi_{\beta }\left(\Xi_{v}|\mu_{\beta },\sigma_{\beta }\right)},\left(\xi =1,2,\cdots ,\vartheta \right),$$

where $\gamma_{\xi \beta }$ is the posteriori probability; ϑ is the total number of samples for the βth cluster.

For the second step, after obtained $\gamma_{\xi \beta }$, the new values of $\tau_{\beta }$, $\mu_{\beta }$, and $\sigma_{\beta }$ can be calculated by the equations

(13) $$\tau_{\beta }=\frac{\Phi_{\beta }}{\vartheta },$$

(14) $$\mu_{\beta }=\frac{1}{\Phi_{\beta }}\sum_{\xi =1}^{\vartheta }\gamma_{\xi \beta }\Xi_{v},$$

(15) $$\sigma_{\beta }=\sum_{\xi =1}^{\vartheta }\gamma_{\xi \beta }\left[\Xi_{v}-\mu_{\beta }\right]{\left[\Xi_{v}-\mu_{\beta }\right]}^{T},$$

If we assume that all the elements of $\theta_{1}$ defined in Equation (9) are independent of each other, repeating the abovementioned two steps, the log-likelihood estimation is utilized to estimate the parameters τ, μ, and σ by maximizing the following objective function.

(16) $$\varsigma \left(\tau ,\mu ,\sigma \right)=\ln\left(\prod_{v=1}^{m}F\left(\Theta_{\vartheta }\right)\right)=\sum_{v=1}^{m}\ln\left(\sum_{\beta =1}^{\alpha }\tau_{\beta }\Phi_{\beta }\left(\Xi_{v}|\mu_{\beta },\sigma_{\beta }\right)\right),$$

With the GMM described above, the damage features projected in the direction of principal components are separated into different clusters. Under each cluster, all the projected damage features satisfy the Gaussian probability distribution. Therefore, the novel detection method based on the Mahalanobis distance can be applied to detect bridge damage.

### 2.3. Procedure of the Proposed Hybrid Method

Following the contents described in the previous two sections, all the damage features are projected in the direction of principal components and the direction of nonprincipal components. For the healthy state of bridge, the residuals of the projected damage features are defined as

(17) $$\begin{array}{l} \gamma_{h1}=\left\{\gamma_{h1,1},\gamma_{h1,2},\cdots ,\gamma_{h1,\beta },\cdots ,\gamma_{h1,\;\alpha }\right\}\\ \gamma_{h1,\beta }=\theta_{h1,\beta }-{\bar{\theta }}_{h1,\beta } \end{array}$$

(18) $$\gamma_{h2}=\theta_{h2}-{\bar{\theta }}_{h2},$$

where $\theta_{h1}$ and $\theta_{h2}$ represent the projected damage features $\theta_{1}$ and $\theta_{2}$ under the healthy state of bridge, respectively; $\gamma_{h1}$ and $\gamma_{h2}$ are the residuals of the projected damage features $\theta_{h1}$ and $\theta_{h2}$, respectively; the subscript h represents the healthy state of bridge; ${\bar{\theta }}_{h1,\beta }$ is the mean value of $\theta_{h1,\beta }$; and ${\bar{\theta }}_{h2}$ is the mean value of $\theta_{h2}$. Using Equation (3), the covariance values of $\gamma_{h1}$ and $\gamma_{h2}$ can be obtained, and with the Mahalanobis distance, the discriminant metrics of residuals are calculated with the following equations.

(19) $$\begin{array}{l} \eta_{h1}=\left\{\eta_{h1,1},\eta_{h1,2},\cdots ,\eta_{h1,\beta },\cdots ,\eta_{h1,\alpha }\right\}\\ \eta_{h1,\beta }=\sqrt{\mathrm{Diag}({\left[\gamma_{h1,\beta }\right]}^{T}{\left[{¶}_{h1,\beta }\right]}^{-1}\left[\gamma_{h1,\beta }\right])} \end{array}$$

(20) $$\eta_{h2}=\sqrt{\mathrm{Diag}({\left[\gamma_{h2}\right]}^{T}{\left[{¶}_{h2}\right]}^{-1}\left[\gamma_{h2}\right])},$$

where $\eta_{h1}$ and $\eta_{h2}$ are the discriminant metrics of $\gamma_{h1}$ and $\gamma_{h2}$, respectively; ${¶}_{h1,\beta }$ is the covariance matrix of $\theta_{h1,\beta }$; ${¶}_{h2}$ is the covariance matrix of $\theta_{h2}$; and Diag(·) represents the operator used to generate the vector based on the diagonal elements of the matrix.

Under the healthy state of the bridge, the threshold values of discriminant metrics are defined as

(21) $$\begin{array}{l} \rho_{1}=\left\{\rho_{h1,1},\rho_{h1,2},\cdots ,\rho_{h1,\beta },\cdots ,\rho_{h1,\alpha }\right\}\\ \rho_{h1,\beta }={\eta_{h1,\beta }|}_{0.95} \end{array}$$

(22) $$\rho_{2}={\eta_{h2}|}_{0.95},$$

where ${\cdot |}_{0.95}$ is the calculation operator used to obtain the median of the 95% confidence level of the probability distribution of the discriminant metrics.

Under the state to be diagnosed for damage, using the same process as described above, the residuals of the projected damage features are defined as

(23) $$\begin{array}{l} \gamma_{d1}=\left\{\gamma_{d1,1},\gamma_{d1,2},\cdots ,\gamma_{d1,\beta },\cdots ,\gamma_{d1,\alpha }\right\}\\ \gamma_{d1,\beta }=\theta_{d1,\beta }-{\bar{\theta }}_{h1,\beta } \end{array}$$

(24) $$\gamma_{d2}=\theta_{d2}-{\bar{\theta }}_{h2},$$

where $\theta_{d1}$ and $\theta_{d2}$ represent the projected damage features $\theta_{1}$ and $\theta_{2}$ under the state to be detected for damage of bridge, respectively; $\gamma_{d1}$ and $\gamma_{d2}$ are the residuals of the projected damage features $\theta_{d1}$ and $\theta_{d2}$, respectively; and the subscript d represents the state to be diagnosed for damage of bridge. Similar to Equations (19) and (20), the discriminant metrics of residuals are calculated by the following equations.

(25) $$\begin{array}{l} \eta_{d1}=\left\{\eta_{d1,1},\eta_{d1,2},\cdots ,\eta_{d1,\beta },\cdots ,\eta_{d1,\alpha }\right\}\\ \eta_{d1,\beta }=\sqrt{\mathrm{Diag}({\left[\gamma_{d1,\beta }\right]}^{T}{\left[{¶}_{h1,\beta }\right]}^{-1}\left[\gamma_{d1,\beta }\right])} \end{array}$$

(26) $$\eta_{d2}=\sqrt{\mathrm{Diag}({\left[\gamma_{d2}\right]}^{T}{\left[{¶}_{h2}\right]}^{-1}\left[\gamma_{d2}\right])},$$

where $\eta_{d1}$ and $\eta_{d2}$ are the discriminant metrics of $\gamma_{d1}$ and $\gamma_{d2}$, respectively.

Assuming the total number of monitoring data samples under the state to be diagnosed is *l*, $\eta_{d1}$ and $\eta_{d2}$ are vectors consisting of *l* elements, e.g., $\eta_{d2}$ is described as

(27) $$\eta_{d2}=\left\{\eta_{d2}^{1},\eta_{d2}^{2},\cdots ,\eta_{d2}^{p},\cdots ,\eta_{d2}^{l}\right\}\left(p=1,2,\cdots ,l\right),$$

Then, the discriminant factor is defined as

(28) $$\{\begin{array}{c} \begin{array}{cc} z=1 & \left(\eta_{d1,\beta }\geq \rho_{1}\right)\mathrm{or}\left(\eta_{d2}^{p}\geq \rho_{2}\right) \end{array}\\ \begin{array}{cc} z=0 & \left(\eta_{d1,\beta }<\rho_{1}\right)\&\left(\eta_{d2}^{p}<\rho_{2}\right) \end{array} \end{array},$$

where $\eta_{d2}^{j}$ is the jth element of $\eta_{d2}$, i.e., ${\left\{\eta_{d2}^{1},\eta_{d2}^{2},\cdots ,\eta_{d2}^{j},\cdots ,\eta_{d2}^{l}\right\}}_{1\times l}$. If the value of z is equal to one, the results obtained with the monitoring data are abnormal at this moment; if the value of z is equal to zero, the results obtained with the monitoring data are normal.

**Figure 2:**
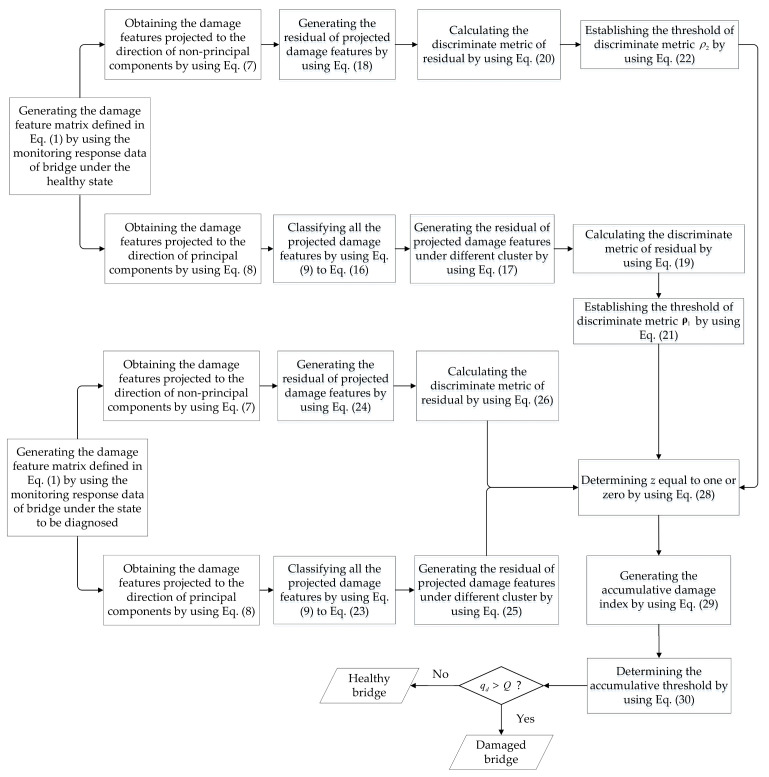
Diagram of the proposed hybrid method for detecting the damage of bridges.

In theory, when structural damage to bridges occurs, abnormal results will be identified by using the proposed method. If the abnormal results calculated by Equation (28) are obtained at some measurement times, the occurrence of structural damage is not guaranteed. When damage occurs to bridges, e.g., the generation of concrete cracks, structural damage is generally not self-healing, and the extent of damage increases over time. Therefore, abnormal results should be treated as a trend change. Based on this point, the cumulative damage index is defined in this study, and it is described as

(29) $$q_{d}=\sum_{p=1}^{l}z_{p},$$

where $z_{p}$, as described in Equation (28), is a sample of a Bernoulli trial with a given success rate *PR* and $q_{d}$ is the cumulative damage index. As shown in Equation (29), the cumulative damage index follows a binomial probability distribution. Therefore, the inverse cumulative distribution function of the binomial distribution can be used to determine the cumulative threshold for damage detection of bridge, which is defined as

(30) $$P_{r}=\sum_{p=0}^{Q}\frac{l!}{p!\cdot \left(l-p\right)!}PR^{p}{\left(1-PR\right)}^{l-p},$$

where P is the probability that there are *Q* successes in *l* trials based on the given success rate *PR*, such as 0.95. The value *Q* is the threshold for the damage decision. If the value of the cumulative damage index $q_{d}$ is larger than the value of *Q*, the bridge is believed to be in a damage state, and vice versa. The entire procedure of the proposed hybrid method is shown in Figure 2.

## 3. Numerical Example

### 3.1. Description of the Numerical Bridge-Like Model

**Figure 3:**
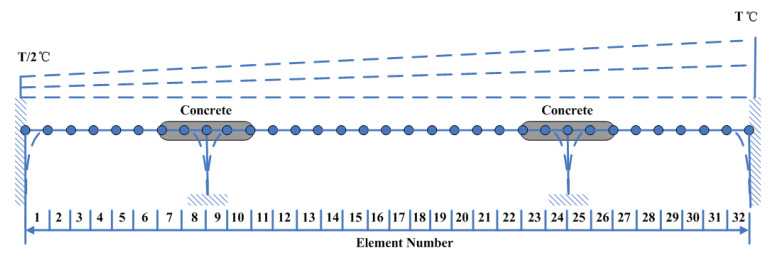
Simulated bridge-like structure.

**Figure 4:**
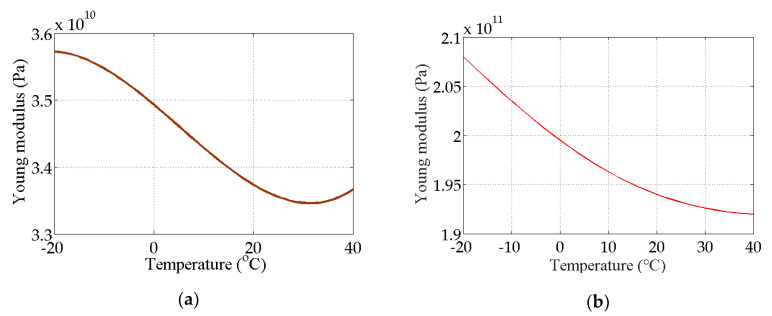
Modulus of elasticity as a function of temperature: (**a**) concrete; (**b**) steel.

**Figure 5:**
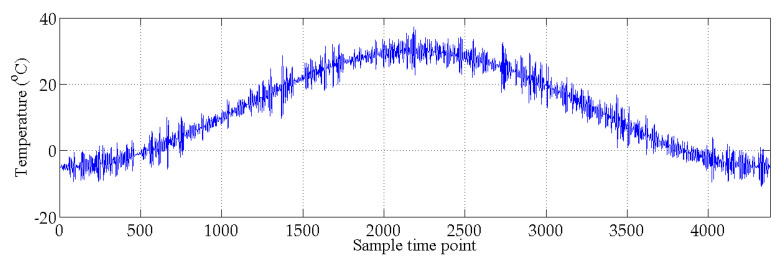
Simulated temperature variations.

**Figure 6:**
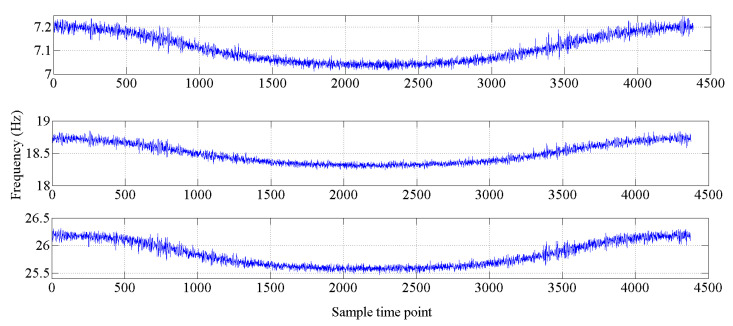
Changes in the natural frequencies (mode 1 to mode 3).

**Figure 7:**
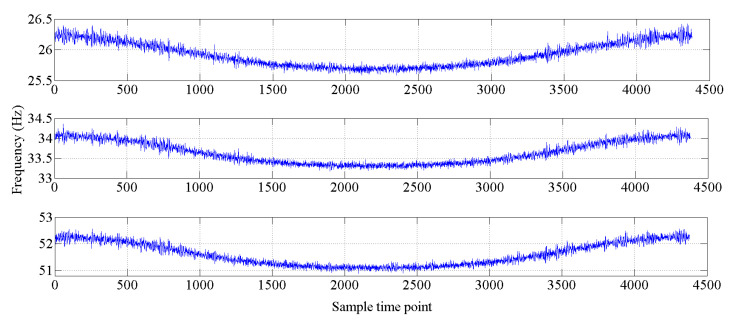
Changes in the natural frequencies (mode 4 to mode 6).

The bridge-like structure introduced by Yan et al. [37] is depicted in Figure 3; this structure has three spans and is discretized with 32 equal-size beam elements. The modules of elasticity of the materials are temperature dependent according to Figure 4, as in [37]. The first six modes of natural frequencies are used to form the damage features. The values of these frequencies are contaminated with Gaussian noise with a standard deviation equal to 5% of the frequency of interest. In this study, to make the simulated variation of air temperature close to the real condition, the annual air temperature changing and the daily air temperature fluctuation are simultaneously considered during the simulation process. The annual air temperature variation is simulated by extending the changing trend of environmental temperature monitoring data which are described in Section 4. The changing range of simulated air temperature is also similar to the results of measured data. The daily air temperature fluctuation is simulated by considering the daily temperature difference and the daily temperature changing trend. The daily temperature difference is defined as 10 °C, and the daily temperature changing trend is simulated by using the sinusoidal function, which simulates the temperature changing from 6:00 a.m. to 6:00 a.m. the next day. The simulated environmental temperature is shown in Figure 5. The changes in the first six modes of the natural frequencies are shown in Figure 6 and Figure 7. As shown in these figures, the correlation between natural frequencies and the environmental temperature is obvious. We use this example to compare the damage detection performance of the proposed hybrid method with that of the PCA-based method.

### 3.2. Comparison of the Performance Levels of the PCA-Based Method and the Proposed Hybrid Method

**Figure 8:**
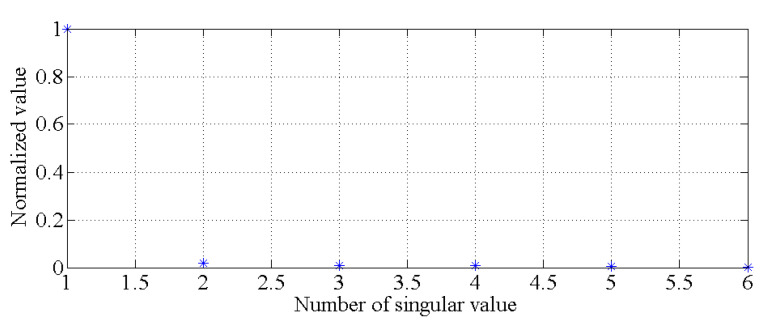
Singular values for determining the principal components.

**Figure 9:**
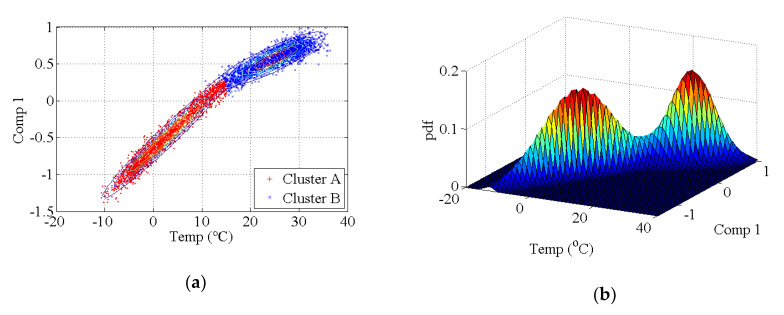
Results of principal component classification by using the GMM. (**a**) two-dimensional results, (**b**) three-dimensional results.

Using the data for the first six modes of the natural frequencies of this bridge-like structure, the singular values of the covariance matrix described in Equation (3) are obtained by using singular value decomposition. As shown in Figure 8, it is obvious that there is only one principal component. With the GMM, the results of the cluster analysis are obtained, as shown in Figure 9. All the natural frequency data shown in Figure 6 and Figure 7 are applied to generate the healthy state model of this bridge-like structure, i.e., to generate $\theta_{1}$ and $\theta_{2}$. Additionally, other data (each mode of a frequency is simulated for 720 samples) are simulated for different cases to compare the damage detection performance of the PCA-based method with that of the proposed hybrid method. A total of three cases are considered, as described in Table 1.

**Figure 10:**
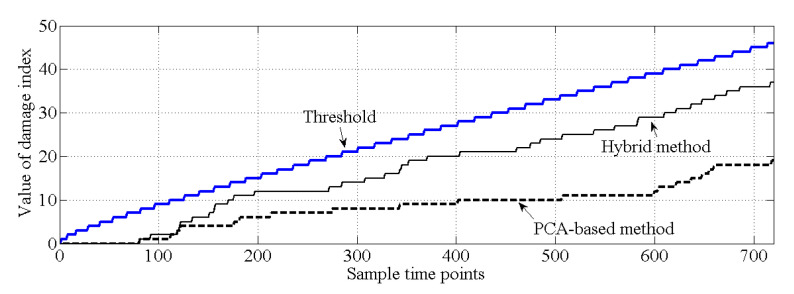
Comparison of the results of damage detection (case 1).

**Figure 11:**
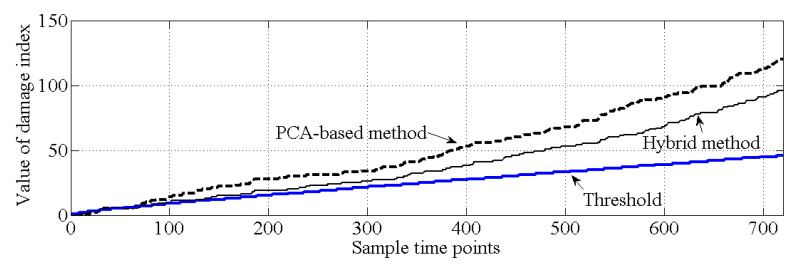
Comparison of the results of damage detection (case 2).

**Figure 12:**
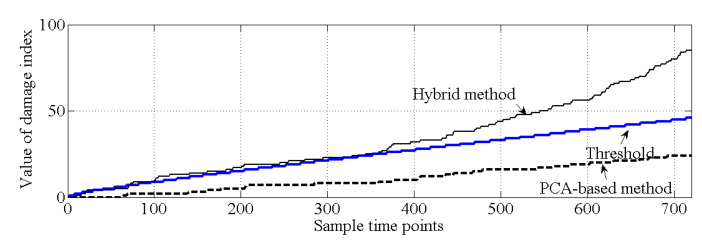
Comparison of the results of damage detection (case 3).

As shown in Figure 10, for the healthy case, both the PCA-based method and the proposed hybrid method make the correct decision because all the values of the cumulative damage index are lower than the threshold. For case 2, it is obvious that the values of the cumulative damage index are larger than the threshold at the first sampling point for both methods. Case 2 is similar with the damage case described in [37], and the result of PCA-based method, shown in Figure 11, is similar with the result obtained by [37]. Notably, both methods can effectively detect structural damage. However, the PCA-based method is more sensitive to the damage that occurred at element 17 than is the hybrid method. Moreover, in case 3, the PCA-based method cannot detect the damage that occurred at element 7. In this case, most of the information related to the damage that occurred at element 7 should exist in the principal component $\theta_{1}$, and this information is deleted by the PCA-based method. For the hybrid method, no information related to damage at element 7 is deleted; thus, the hybrid method can work well for case 3, shown as Figure 12.

## 4. Example of an Actual Bridge

**Figure 13:**
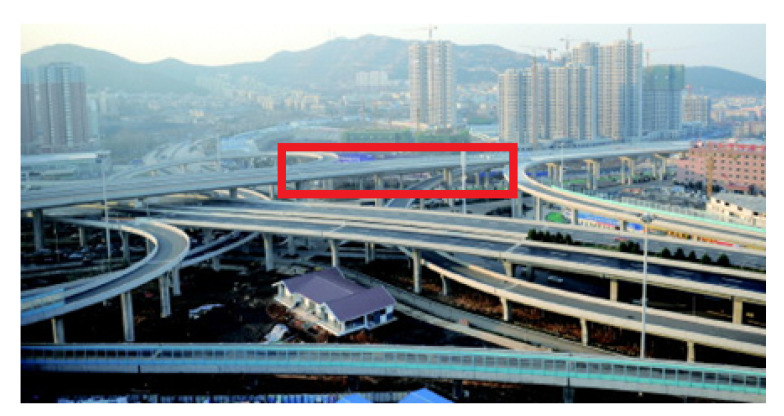
Photograph of an actual bridge.

**Figure 14:**
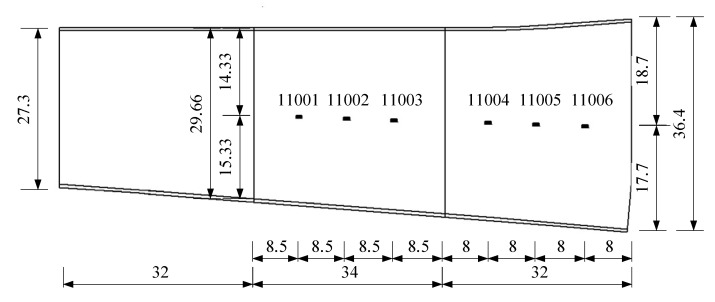
Arrangement of acceleration sensors (unit: m).

**Figure 15:**
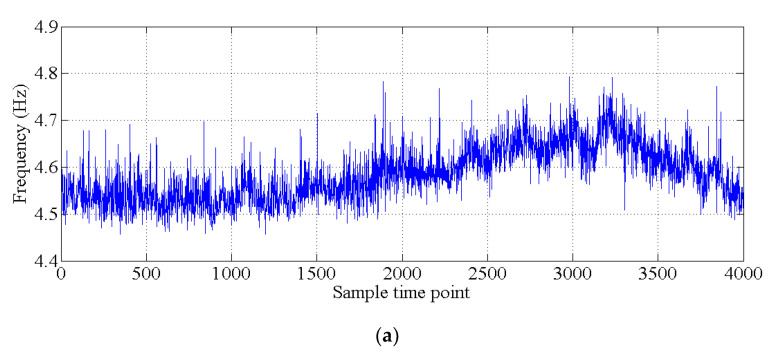

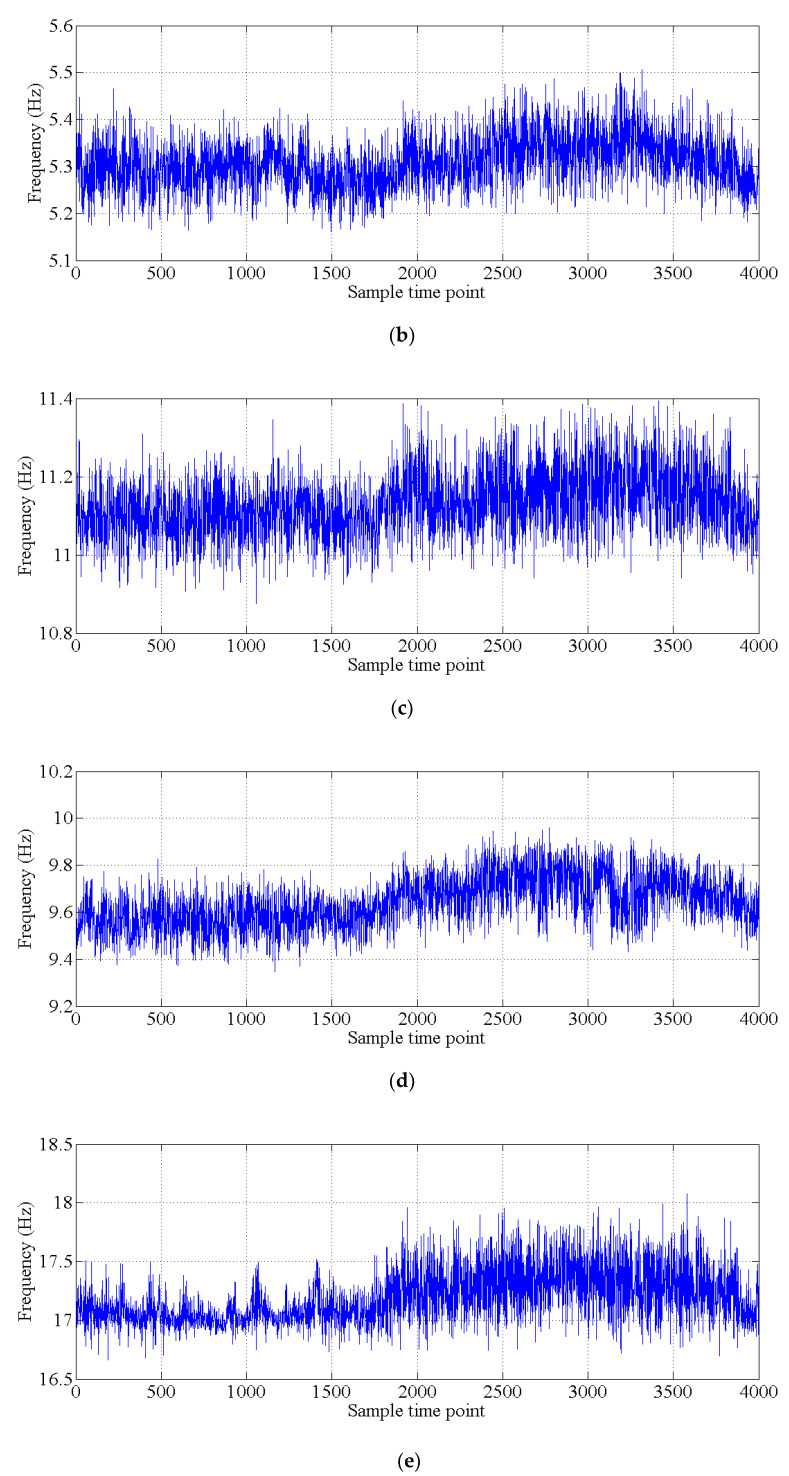

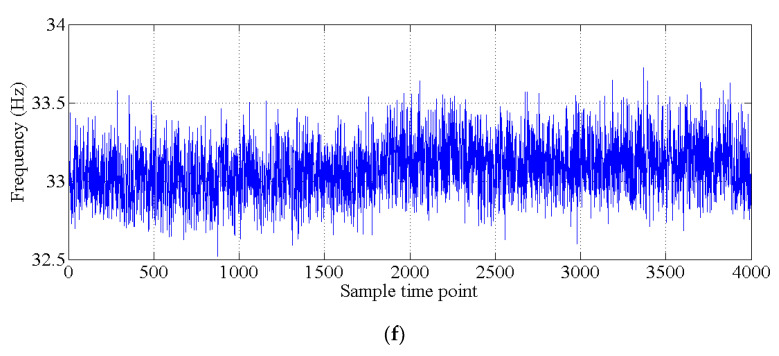
Natural monitoring frequencies: (**a**) mode 1; (**b**) mode 2; (**c**) mode 3; (**d**) mode 4; (**e**) mode 5; (**f**) mode 6.

**Figure 16:**
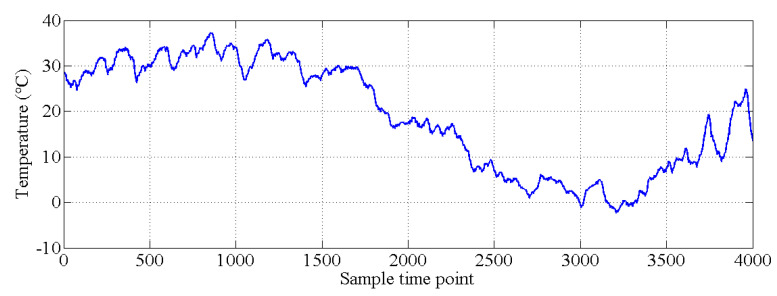
Environmental temperature monitoring data.

In this section, the natural frequencies monitoring data obtained from an actual bridge are applied to verify the performance of the proposed hybrid method. This bridge is healthy, and there is no known damage to the structure, so only the damage detection performance of the proposed hybrid method is verified. As shown in Figure 13, a three-span continuous-beam bridge [50], which is one part of an interchange bridge, is taken as an example. The SHM system of this bridge began operation in October 2015. The acceleration monitoring data for the first year are utilized to demonstrate the effectiveness of the proposed hybrid method. The arrangement of six acceleration sensors is shown in Figure 14. The hourly acceleration data are used to identify the natural frequencies of the bridge by using the eigensystem realization algorithm (ERA) [51]. The variations in the natural monitoring frequencies are shown in Figure 15. Additionally, the changes in the environmental temperature monitoring data are shown in Figure 16. Each point on the curve shown in Figure 16 represents the average environmental temperature in each hour. By comparing the results in the above two figures, it is obvious that the natural frequencies of this bridge exhibit a negative correlation with the environmental temperature.

**Figure 17:**
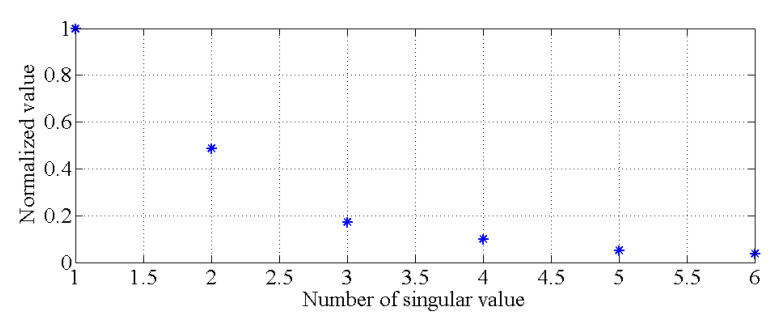
Singular values for determining the principal components of an actual bridge.

**Figure 18:**
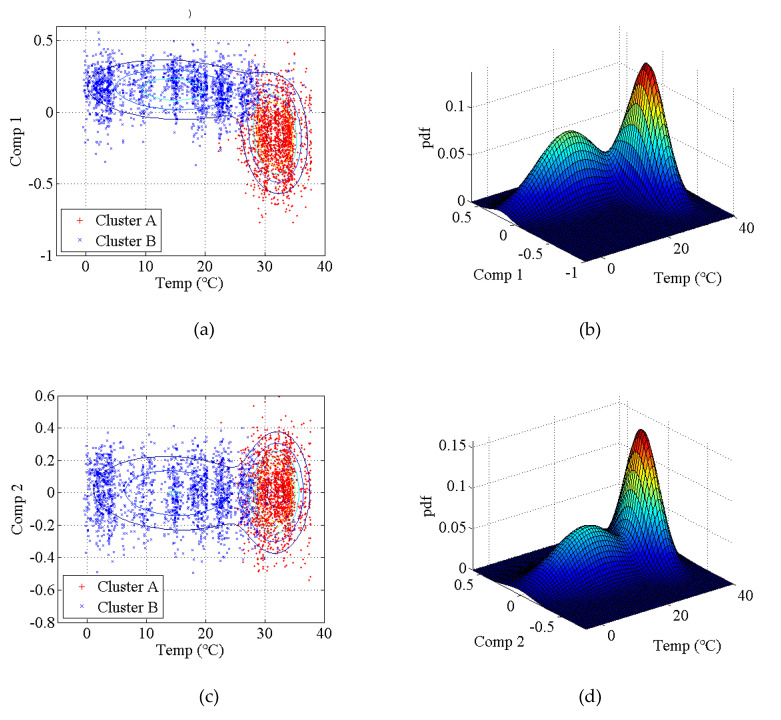

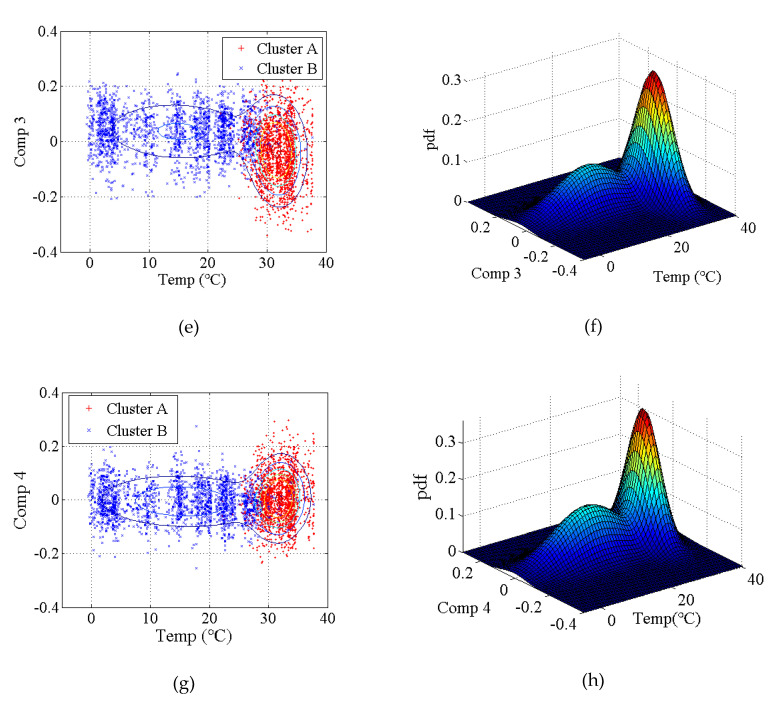
Results of the cluster analysis of principal components for an actual bridge: (**a**) two-dimensional results of the first principal component; (**b**) three-dimensional results of the first principal component; (**c**) two-dimensional results of the second principal component; (**d**) three-dimensional results of the second principal component; (**e**) two-dimensional results of the third principal component; (**f**) three-dimensional results of the third principal component; (**g**) two-dimensional results of the fourth principal component; (**h**) three-dimensional results of the fourth principal component.

The covariance matrix of natural monitoring frequency data is generated by using all the monitoring data shown in Figure 16. Then, the singular values of the abovementioned covariance matrix are obtained by using singular value decomposition. As shown in Figure 17, the first four components are selected as the principal components. With the GMM, the results of cluster analysis are obtained, as shown in Figure 18. The results of cluster analysis show that all the natural frequencies are generally classified into two clusters, which correspond to the winter and summer. The relationship between the natural frequencies of this bridge and the environmental temperature is nonlinear.

**Figure 19:**
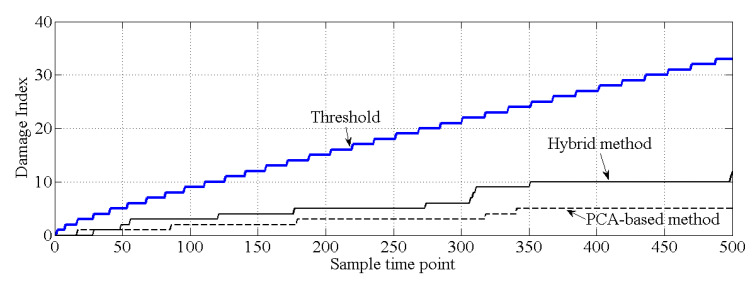
Results of damage detection for an actual bridge.

Except for the last 500 samples of natural monitoring frequency data shown in Figure 15, all the other data are applied to generate the healthy state model of this bridge, i.e., to generate $\theta_{1}$ and $\theta_{2}$. The last 500 samples are utilized to verify whether the proposed hybrid method will make the wrong decision in damage detection. The results of damage detection are shown in Figure 19, and both the PCA-based method and the proposed hybrid method make the correct decision.

## 5. Conclusions

In this study, a hybrid method is proposed to detect the damage of bridges under environmental temperature changes. The following conclusions are drawn from the analysis.

(i) The results of the numerical example show that the PCA-based method may fail to detect damage when the information related to structural bridge damage is mostly stored in the principal components of the covariance matrix composed of damage features because some effective information is deleted by the PCA-based method. The proposed hybrid approach can effectively solve the abovementioned issue.

(ii) The numerical example and example of an actual bridge show that the proposed hybrid method is effective in detecting bridge damage under environmental temperature changes.

(iii) The results of cluster analysis for the actual bridge example show that the GMM is effective for classifying the natural monitoring frequency data of actual bridges, and the relationship between the natural frequencies of actual bridges and the environmental temperature is not always linear.

(iv) Compared with the PCA-based method, the proposed hybrid method requires environmental temperature monitoring data to classify the principal components. If these environmental temperature data cannot be supplied by the SHM system of a bridge, the application conditions of the proposed hybrid method will not be satisfied.

## Figures and Tables

**Table 1:** Descriptions of all cases for the numerical example.

Case Number	Description of Case
Case 1	Healthy structure
Case 2	Damaged structure with 20% reduction in stiffness at element 17
Case 3	Damaged structure with 20% reduction in stiffness at element 7
